# Hydrogen Production from Methanol Steam Reforming over Fe-Modified Cu/CeO_2_ Catalysts

**DOI:** 10.3390/molecules29163963

**Published:** 2024-08-22

**Authors:** Grzegorz Słowik, Marek Rotko, Janusz Ryczkowski, Magdalena Greluk

**Affiliations:** Department of Chemical Technology, Institute of Chemical Sciences, Faculty of Chemistry, Maria Curie-Sklodowska University in Lublin, 3 Maria Curie-Skłodowska Square, 20-031 Lublin, Poland; marek.rotko@mail.umcs.pl (M.R.); janusz.ryczkowski@mail.umcs.pl (J.R.); magdalena.greluk@mail.umcs.pl (M.G.)

**Keywords:** copper–iron catalysts, ceria support, steam reforming of methanol, hydrogen production

## Abstract

Fe-modified Cu catalysts with CeO_2_ support, prepared by the impregnation method, were subjected to physicochemical analysis and catalytic tests in the steam reforming of methanol (SRM). Physicochemical studies of the catalysts were carried out using the XRF, TEM, STEM-EDS, XRD, TPR and nitrogen adsorption/desorption methods. XRD, TEM studies and catalytic tests of the catalysts were carried out at two reduction temperatures, 260 °C and 400 °C, to determine the relationship between the form and oxidation state of the active phase of the catalysts and the catalytic properties of these systems in the SRM. Additionally, the catalysts after the reaction were analysed for the changes in the structure and morphology using TEM methods. The presented results show that the composition of the catalysts, morphology, structure, form and oxidation state of the Cu and Fe active metals in the catalysts and the reaction temperature significantly impact their activity, selectivity and stability in the SRM process. The gradual deactivation of the studied catalysts under SRM conditions could result from the forming of carbon deposits and/or the gradual oxidation of the copper and iron phases under the reaction conditions.

## 1. Introduction

The increase in the demand for electricity and the shrinking resources of fossil fuels affect the growing interest in obtaining energy from alternative and renewable sources. One of the most promising renewable energy carriers is hydrogen, which, apart from the highest energy value per mass unit, produces only water when burned. Hydrogen is very often considered as a fuel of the future. Unlike fossil fuels, it is a clean energy source that does not contribute to the emission of such undesirable compounds as CO_2_, CO, SO_2_, NO_x_, particulate matter or hydrocarbons. Increasingly stringent environmental protection regulations result in a growing interest in using hydrogen as a fuel to power fuel cells, which is converted into useful electricity that can be used in stationary devices or to power electric vehicles. One of the many methods proposed for hydrogen production, the most popular and energy-efficient catalytic process, is the steam reforming of methanol (SRM). When the methanol reacts with steam, under the most favourable SRM process conditions, one mole of methanol can produce as many as three moles of hydrogen. Moreover, methanol as a hydrogen source belongs to relatively readily available raw materials and can be obtained from renewable energy sources [1].

In the SRM process, Equation (1), two main reactions are most often taken into account, methanol decomposition, Equation (2), and the water–gas shift (WGS), Equation (3), as follows [2,3]:

CH_3_OH + H_2_O → CO_2_ +3H_2_ (1)

CH_3_OH → CO + 2H_2_ (2)

CO + H_2_O → CO_2_ +H_2_ (3)

However, the hydrogenation of CO, Equation (4), or CO_2_, Equation (5), is also possible according to the reactions shown below [4]:

CO + 3H_2_→ CH_4_ + H_2_O (4)

CO_2_ + 4H_2_→ CH_4_ + 2H_2_O (5)

The SRM process, influenced by factors such as the methanol-to-water ratio, temperature and the catalytic properties of the catalyst, can lead to the formation of undesirable products. To ensure the complete and selective conversion of methanol with steam, developing a specific and efficient catalyst system becomes imperative. An effective SRM catalyst should exhibit good activity, selectivity to the main reaction products (H_2_ and CO_2_) and long-term stability under process conditions [1]. This underscores the critical role of catalysts in the efficient production of hydrogen.

Catalysts containing noble metals, such as Pt and Pd, as the active phase show higher activity in the SRM than catalysts based on Cu [5,6,7,8,9,10]. However, the high price of noble metal-containing catalysts limits their potential application in the SRM. Therefore, copper and metals of 8–10 periodic table groups are more often studied for the SRM due to their high catalytic efficiency and low cost [11,12,13,14,15,16,17,18,19,20]. Nevertheless, Cu-based catalysts exhibit pyrophoric properties and are deactivated through thermal sintering [20]. One of the solutions to improve the stability of these systems and reduce their costs is to introduce other metals, usually from groups 8–10 of the periodic table, which most often improve the stability and show good selectivity towards hydrogen. Unfortunately, these metals exhibit lower methanol conversion than Cu-based catalysts [21,22].

A promoter-addition approach is often used to prevent the low stability of Cu-based catalysts [22,23,24,25,26,27]. It has been shown that Fe can effectively improve the strength of Cu particles interaction with the support surface in the SRM [23,28,29,30,31]. Fe can also improve Cu dispersion by inhibiting its sintering [30,31]. In turn, the catalytic performance of Cu-based catalysts for the SRM strongly depends on the interaction of Cu with the support and promotor or modifier used [26,32,33,34]. In recent years, the unique features of copper-based catalysts, particularly Cu/CeO_2_, for the steam reforming of methanol have sparked significant research interest. Their high activity, cost-effectiveness and ability to store oxygen, which is crucial for eliminating CO in the WGS reaction, make them a promising avenue for the future [35,36,37]. The role of the copper–cerium interface, with its numerous oxygen vacancies at the surface [38,39], the transformation between Ce^4+^ and Ce^3+^ [39] and the accompanying electron transfer from metallic copper to ceria [36,40], has particularly intrigued scientists. The impressive performance of the Cu/CeO_2_ catalyst under SRM conditions was mainly attributed to the highly dispersed Cu metal particles on the ceria surface [41,42,43], the strong metal–support interaction between the Cu metal and CeO_2_ support [41,42] and the large amounts of oxygen vacancies on the surface [43]. Some authors have indicated that the form of the Cu species influences the Cu/CeO_2_ catalyst activity under SRM conditions, proposing the presence of Cu^0^ and CuO species [42] or Cu^0^ and Cu_2_O species [44] as essential for obtaining a highly effective Cu/CeO_2_ catalyst in the SRM process. Moreover, the difference in the specific morphology and structure of ceria support can influence the lattice planes, oxygen storage and release capacity and metal-to-metal interactions, affecting the catalytic performance of CuO/CeO_2_ catalysts in the SRM reaction [42,45].

It is important to note that the literature on using Cu-Fe systems in SRM reactions is scarce, with only two papers available. Yu et al. [46] indicated that the 70CuFeO_2_/30CeO_2_catalyst exhibited high activity in the SRM due to the synergistic effect between the CuFeO_2_ and CeO_2_ support. According to Cao et al. [47], the synergistic effects between CuO and Fe_2_O_3_, which favour the reduction and dispersion of CuO in the catalyst, mainly influence the high methanol conversion, high selectivity to H_2_ and deficient CO concentration over the Cu-Fe/ATP (attapulgite) catalyst under SRM conditions.

However, the literature lacks comprehensive information about Cu-Fe/CeO_2_ catalysts and their physicochemical and catalytic relationships. This gap in knowledge underscores the urgent need for further research on Cu-Fe/CeO_2_ catalysts for the steam reforming of methanol. The pressing need for more studies in this area cannot be overstated. The results presented in this work are a significant step towards filling this gap and contributing to the existing knowledge on this subject. Thus, in this work, we present the effect of the active phase’s composition, structure, morphology and oxidation state on the activity, selectivity and stability of the Cu-Fe/CeO_2_ catalysts in the steam reforming of methanol.

## 2. Results and Discussion

### 2.1. Optimisation of the Copper Catalyst Composition—Activity and Selectivity of Cu/CeO_2_ Catalysts in the SRM

**Figure 1:**
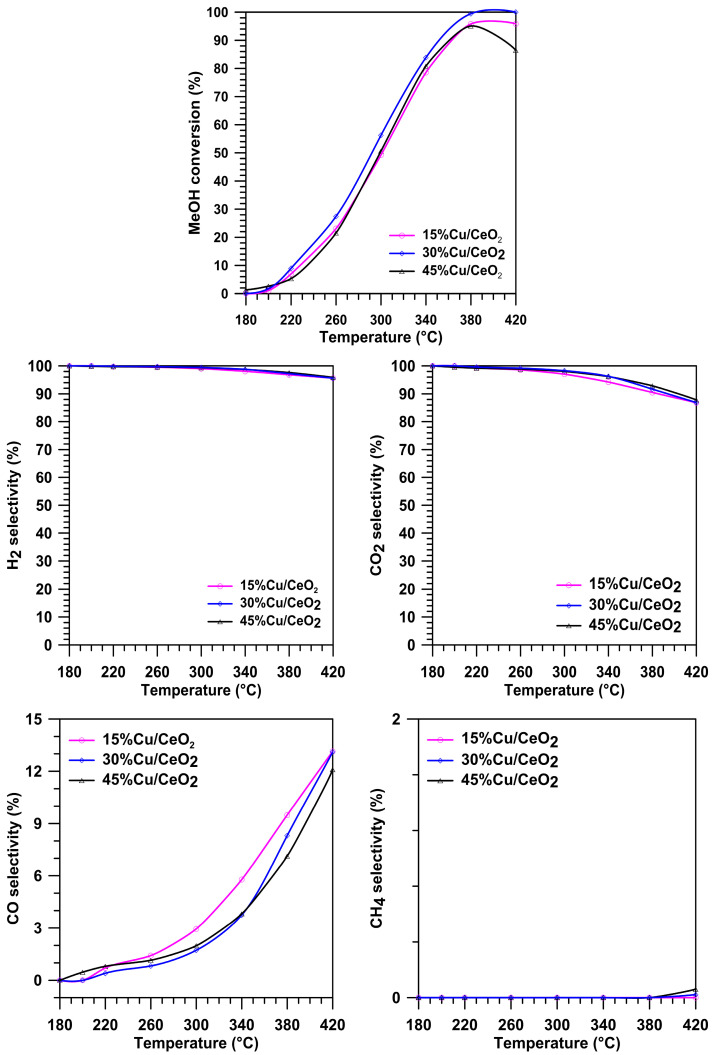
The conversion of MeOH and selectivity to H_2_, CO_2_, CO and CH_4_ on the Cu/CeO_2_ catalysts at different temperatures in the SRM.

To optimise the best catalytic efficiency of the Cu/CeO_2_ system, catalysts containing 15%, 30% and 45% of copper active phase were prepared. Figure 1 shows the dependence of the MeOH conversion and selectivity to the products in the SRM on the reaction temperature over the prepared copper catalysts. With the increase in the reaction temperature, an increase in the MeOH conversion in the process can be observed for all of the studied systems. In the case of all of the catalysts, the increase in the reaction temperature caused a slight decrease in the selectivity to H_2_ (by 5% in the temperature range from 300 to 420 °C) and CO_2_ (by 12% in the temperature range from 260 °C to 420 °C). Moreover, a gradual increase in CO production was also observed with the increase in the reaction temperature of the process for all systems. The most significant increase in the CO production in the SRM process was observed from the temperature of 300 °C, which may be related to a decrease in the efficiency of the WGS reaction in the SRM process. With the increase in the SRM reaction temperature from 380 °C, a small amount of CH_4_was also detected due to the hydrogenation of CO or CO_2_.

Compared to the other samples, the catalyst containing 30% Cu exhibited the highest MeOH conversion in the temperature range from 210 to 420 °C, and the temperature of 380 °C was sufficient to achieve a complete MeOH conversion over this catalyst.

In the case of the 45Cu/CeO_2_ catalyst, the overloading can induce Cu^0^ growth into big particles that lose the methanol conversion properties, whereas low methanol conversion over the 15Cu/CeO_2_ catalyst indicates that, due to low loading, the amount of copper active sites on the CeO_2_ surface was insufficient. Therefore, because the highest methanol conversion was obtained over the 30Cu/CeO_2_ catalyst, it was chosen to contain an optimal Cu content among all of the tested systems.

The optimisation of the active phase composition in the studied copper catalysts was used to prepare a series of bimetallic catalysts based on copper and iron as the active phase, of which the total content was 30%. The prepared bimetallic Co-Fe/CeO_2_ catalysts were marked as 20Cu-10Fe/CeO_2_, 15Cu-15Fe/CeO_2_ and 10Cu-20Fe/CeO_2_, respectively, where the individual numerical values denote the percentages of active phase elements assumed during the preparation. The results presented in this paper were mainly focused on the catalysts with extreme Cu and Fe contents (i.e., 20Cu-10Fe/CeO_2_ and 10Cu-20Fe/CeO_2_) and the 30Cu/CeO_2_ catalyst as the reference.

### 2.2. Qualitative and Quantitative Composition of Elements in the Cu-Fe/CeO_2_ Catalysts

Table 1 presents the XRF analysis of the copper and iron contents (wt.%) in the prepared Cu-Fe catalysts. The expected total metal (Cu + Fe) loading was maintained at 30 wt.%. As can be observed, the measured metal contents were close to the nominal values, confirming that the synthesis method was successful.

### 2.3. Specific Surface Area and Pore Size of Catalysts

The BET data for the support and fresh catalyst are summarised in Table 1, indicating the maintenance of the CeO_2_ mesoporous structures. As expected, the impregnation of the copper and iron precursors reduced the surface area due to the metals’ phases filling the pores or occupying the inner surface of the CeO_2_ supports [48,49,50]. The particles of both iron and/or copper oxides blocked the pore structure of CeO_2_ and did not form the new small pores inside of the large pores, so the S_BET_ decreased remarkably. The exception to this rule is the 10Cu-20Fe/CeO_2_ catalyst, i.e., the sample containing the highest amount of Fe. The increased S_BET_ and decreased pore size compared to the CeO_2_ support indicated that, indeed, the particles of metal oxides entered into the original large pores and deposited on the inner surface of CeO_2_ to form the small pores [51]. However, it could not also be excluded that the increase in S_BET_ was due to the introduction of iron oxides, which prevented the structural collapse of CeO_2_ during calcination. The decrease in the S_BET_ after loading a higher amount of copper oxides and a lower amount of iron oxide can be attributed to the fact that partial CeO_2_ was sintered and grown during the catalyst calcination process [52,53,54].

### 2.4. Phase Composition and Average Crystallite Size

#### 2.4.1. XRD

**Figure 2:**
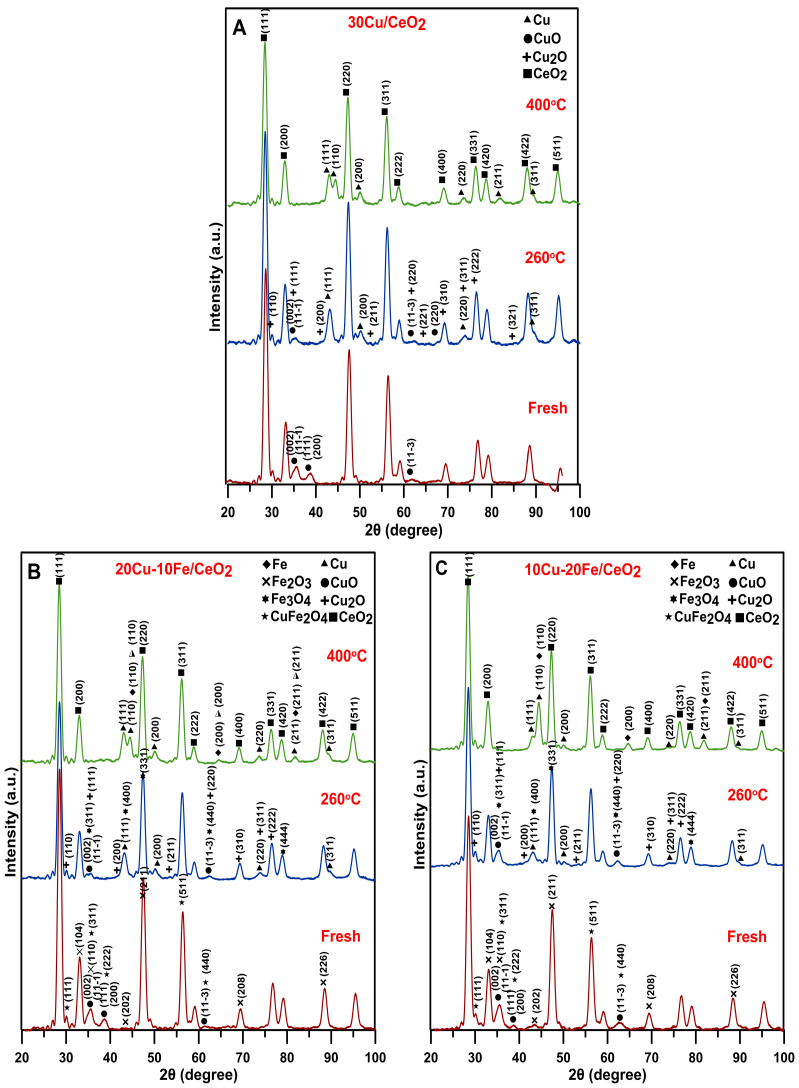
XRD diffractograms of fresh and insitu reduced (**A**) 30Cu/CeO_2_, (**B**) 20Cu-10Fe/CeO_2_and (**C**) 10Cu-20Fe/CeO_2_ catalysts at 260/400 °C made in the angle 2θ from 20° to 100°.

The bulk structure of the Cu/CeO_2_ and Cu-Fe/CeO_2_ catalysts in the fresh form (after calcination) and after reduction at 260 °C and 400 °C was characterised by the XRD method, and the diffractograms are shown in Figure 2A–C. In the case of all of the catalysts, the prominent peaks come from the CeO_2_ support_,_ which is the most abundant and constitutes the primary phase.

The fresh 30Cu/CeO_2_ catalyst (Figure 2A) exhibited peaks from the CuO phase. After reducing the catalyst at 260 °C, the copper active phase was significantly reduced to metallic copper (Cu^0^). However, low-intensity peaks from copper oxides (both CuO and Cu_2_O) suggest the difficulty in reducing bulk copper oxide species. Their complete reduction to metallic copper was observed only after the reduction with hydrogen at 400 °C. In the case of the catalysts containing both copper and iron (Cu-Fe/CeO_2_) (Figure 2B,C), it can be observed that, in their fresh form, the copper existed in the form of CuO and the iron occurred in the form of Fe_2_O_3_ and/or CuFe_2_O_4_ spinel. After reducing the catalysts at 260 °C, some changes in the copper phase and a slight change in the iron phase were observed in the studied catalysts.

The active copper phase was reduced mainly to metallic copper, as indicated by the intense diffractogram peak at 2θ = 43.3°. Nevertheless, a small amount of copper in the form of CuO and Cu_2_O may also be present, but this is not clear because the peaks from the CuO and Cu_2_O phases show a low intensity and are additionally located in very similar positions as the peaks from the Fe_3_O_4_ and Cu^0^ phases. After reducing the Cu-Fe/CeO_2_ catalysts at 400 °C, the active phase consisted of metallic copper (Cu^0^) and metallic iron (Fe^0^). The formation of the Cu-Fe alloy in the studied bimetallic Cu-Fe/CeO_2_ systems can be excluded under these reduction conditions. Firstly, copper and iron are slightly soluble in each other, and a two-phase region can be observed in the Cu-Fe phase diagram in a wide range of concentrations [55]. In addition, the Cu-Fe alloy belongs to a typically metastable immiscible alloy system, exhibiting a metastable immiscible gap under the liquidus, and there is a massive trend of severe segregation or easy phase separation during solidification [56].

#### 2.4.2. TEM

##### TEM Studies of Fresh Cu-Fe Catalysts

Phase analysis based on HRTEM combined with FFT and the STEM-EDS chemical analysis of fresh (after calcination) catalysts is presented in Supplementary Materials (Figures S1 and S2).

##### TEM and STEM-EDS Studies of Reduced Catalysts

**Figure 3:**
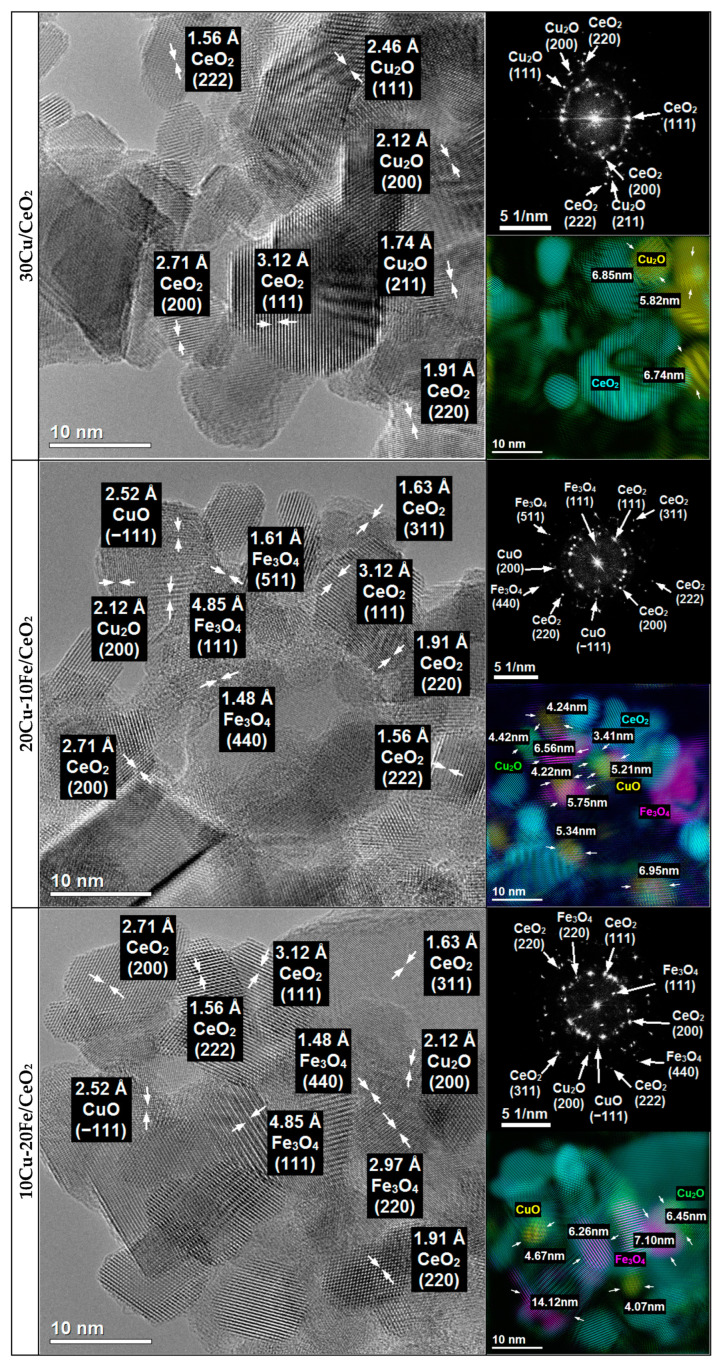
HRTEM images and FFT with phase identification of 30Cu/CeO_2_, 20Cu-10Fe/CeO_2_ and 10Cu-20Fe/CeO_2_ catalysts reduced at 260 °C.

**Figure 4:**
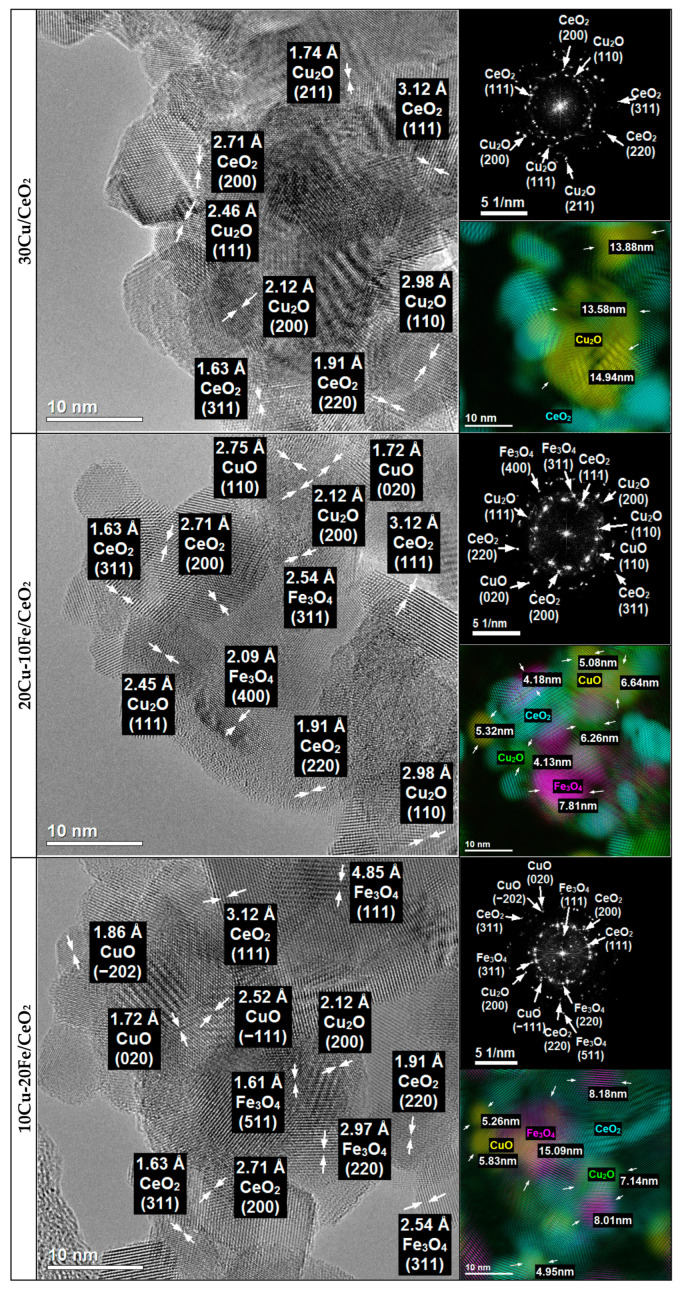
HRTEM images and FFT with phase identification of 30Cu/CeO_2_, 20Cu-10Fe/CeO_2_ and 10Cu-20Fe/CeO_2_ catalysts reduced at 400 °C.

The 30Cu/CeO_2_catalyst and the bimetallic 20Cu-10Fe/CeO_2_ and 10Cu-20Fe/CeO_2_ catalysts after two different reduction temperatures at 260 °C and 400 °C were analysed for their morphology, structure and chemical composition using TEM (Figure 3 and Figure 4) and STEM-EDS (Figures S3 and S4) methods.

**Figure 5:**
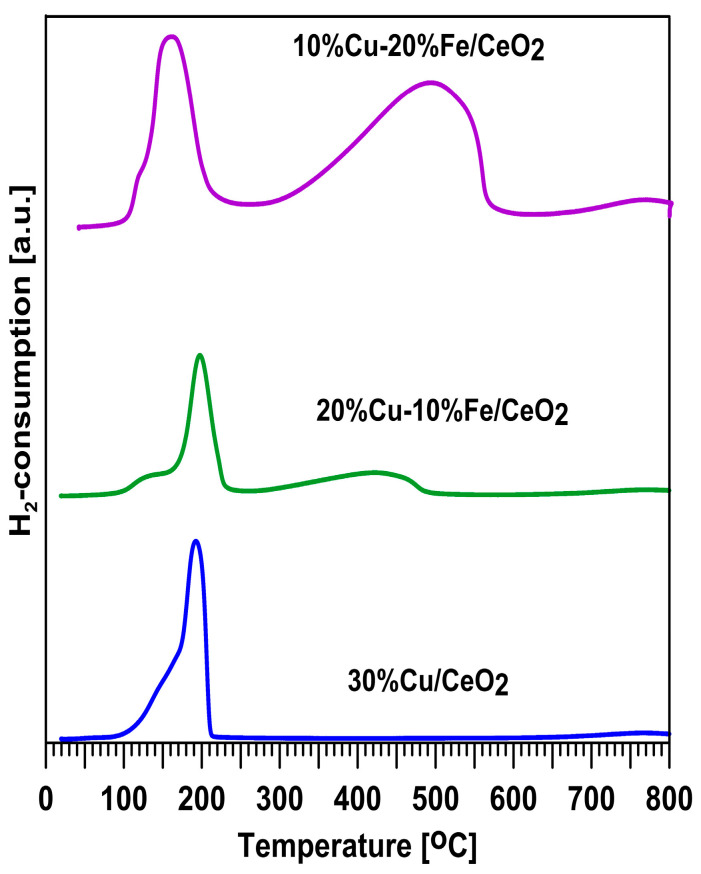
TPR analysis of Cu-Fe/CeO_2_ catalysts.

The phase identification carried out for all of the catalysts after reduction at 260 °C and 400 °C based on HRTEM imaging and FFT (Figure 3 and Figure 4) showed that the majority of the crystalline phase was the CeO_2_ support phase, which was identified based on the interplanar distances of 3.12 Å, 2.71 Å, 1.91 Å, 1.63 Å and 1.56 Å, corresponding to the lattice planes (111), (200), (220), (311) and (222), respectively (Figure 5).

Because copper and iron strongly oxidise under atmospheric conditions, both phases could be oxidised during transport from the reduction reactor to the electron microscope. Therefore, in contrast to the results obtained by XRD, the TEM microscopy identified copper phases only in oxide form. Since the XRD results were performed under insitu conditions, these results would appear to be more reliable. However, phase identifications were also made using TEM microscopy and are described in detail in Supplementary Materials.

The crystallite sizes of the Cu and Fe phases based on the TEM measurement for all of the catalysts are listed in Table 2. In the case of the 30Cu/CeO_2_ catalyst, the Cu_2_O crystallite size after reduction at 400 °C is much larger than at 260 °C. This means that the reduction of the 30Cu/CeO_2_ catalyst at the temperature of 400 °C caused the sintering of the copper phase crystallites.

Also, the STEM-EDS analysis (Figures S3 and S4) confirms the better dispersion of the copper active phase on the CeO_2_ support for the catalyst reduced at 260 °C than at 400 °C. However, it is important to note that agglomerates of Cu_2_O crystallites were still observed, even for the sample reduced at 260 °C. In both of the bimetallic systems, the reduction temperature did not influence the size of the Cu_2_O, CuO and Fe_3_O_4_ (or CuFe_2_O_4_) crystallites (Table 2). Also, the EDS maps of the bimetallic catalysts (Figures S3 and S4) reveal the good dispersion of both the copper and iron phases on the surface of the CeO_2_ support, independent of the temperature of the reduction. However, as can be seen, some of the copper active phase crystallites sintered during the Cu-Fe/CeO_2_ catalyst reduction at 400 °C.

Moreover, independently from the reduction temperature, the crystallites of the Cu phases in the bimetallic Cu-Fe samples are smaller than those in the monometallic 30Cu/CeO_2_ catalyst (Table 2). This may indicate that iron prevented copper crystallites from sintering and forming larger ones. Indeed, on the EDS maps of Cu+Fe and Cu+Fe+Ce (Figures S3 and S4), it can be observed that smaller Cu crystallites occur in places with more Fe clusters, while larger Cu crystallites arise in areas with few Fe crystallites. Hence, it can be concluded that Fe can prevent the sintering of Cu crystallites by blocking the movement of these crystallites on the surface of the CeO_2_ support, thus contributing to the formation of the smaller Cu crystallites. This is related to the immiscible interaction between Cu and Fe, where Fe compounds can suppress the sintering of Cu particles [43]. On the other hand, the crystallite size of copper phases in the case of the Cu-Fe samples can be smaller because these catalysts contain much less copper than the 30Cu/CeO_2_ catalyst. This seems highly likely, since the microscopic analysis confirms that a decrease in the amount of copper and a simultaneous increase in the amount of iron resulted in a reduction of the crystallite size of the copper and an increase in the crystallite size of the iron.

HRTEM imaging combined with FFT (Figure 3 and Figure 4) and the STEM-EDS elemental analysis (Figures S3 and S4) also showed that, in the case of fresh catalysts, and after their reduction at 260 °C, the Cu_2_O crystallites were much more covered by the CeO_2_ support, whereas the Cu_2_O crystallites of the catalysts after reduction at 400 °C were much better exposed. This suggests that the catalysts’ reduction temperature could impact the efficiency of the methanol conversion in the SRM process. Despite their large size, copper phase crystallites after the catalysts’ reduction at 400 °C are better exposed to the SRM process reagents. This means that they could operate much more effectively in the process than smaller copper crystallites of catalysts reduced at 260 °C, which are less exposed and more covered by the crystallites of the CeO_2_ support.

### 2.5. H_2_-TPR Studies

Figure 5 shows the reducibility of the Cu-Fe catalysts in the presence of hydrogen with an increasing reduction temperature. On all of the TPR profiles, a relatively narrow region of low-temperature reduction of about 100–220 °C can be observed without a clear separation in the reduction stages. Additionally, in the case of the Cu-Fe systems, a broad region of high-temperature reduction without a clear separation in the reduction stages in the temperature range of 300–550 °C is observed. For all of the analysed samples, the reduction begins at 100 °C. With the increase in the iron content, the intensity of the reduction peak in the temperature range of 100–220 °C decreases, while the intensity of the reduction peak in the temperature range of 300–550 °C increases. Thus, it can be concluded that the low-temperature peak corresponds to the reduction of the oxygen form of copper to the metallic copper, Cu^0^ [40,57,58,59,60], and the high-temperature peak corresponds to the reduction of iron oxide forms to the metallic iron, Fe^0^, [61] and the reduction of CuFe_2_O_4_ spinel to metallic copper and metallic iron [62]. Moreover, all of the catalysts exhibit well-visible reduction peaks starting at 680 °C, which can be attributed to the reduction of the bulk CeO_2_ [46].

### 2.6. Activity, Selectivity and Stability of Cu-Fe/CeO_2_ Catalysts Reduced at 260/400 °C during an Isothermal Test at 260 °C in the SRM

**Figure 6:**
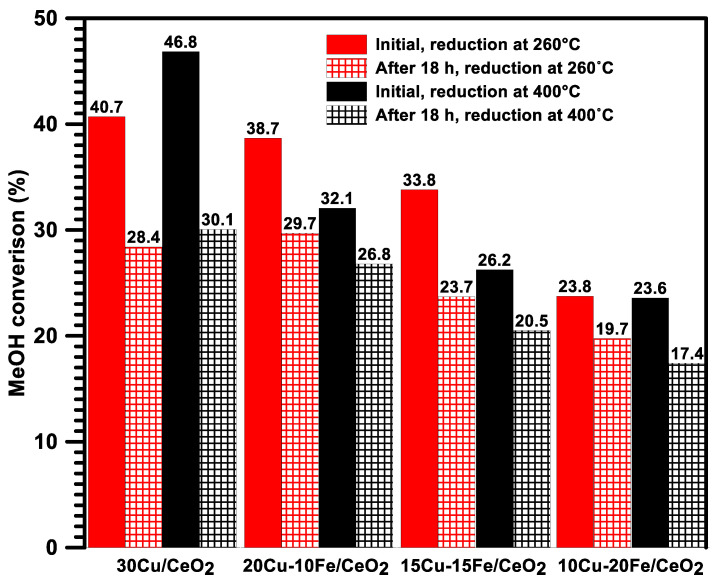
Comparison of the initial and final conversion of MeOH on the Cu/CeO_2_ and Cu-Fe/CeO_2_ catalysts reduced at 260/400 °C in the steam reforming of methanol at 260 °C.

Figure 6 shows the initial and final (after 18 h) conversion of the 30Cu/CeO_2_ and Cu-Fe/CeO_2_ catalysts reduced at 260 °C or 400 °C in the steam reforming of methanol at 260 °C. In the initial phase of the catalytic tests, the highest conversion of all of the studied systems was observed on the 30Cu/CeO_2_ catalyst, regardless of the catalysts’ reduction temperature. With the decrease in the copper content, a decrease in the methanol conversion was observed. Therefore, the 10Cu-20Fe/CeO_2_ catalyst was the least active in the SRM process. Nevertheless, the presence of iron influences the stability of copper-based catalysts, which increases with the increase in the iron content. Therefore, the most significant decrease in the methanol conversion over time was observed for the 30Cu/CeO_2_ catalyst, whereas, in the case of Cu-Fe/CeO_2_ samples, it was considerably minor. These results indicate that adding iron and reducing the copper content in the copper catalyst may have deteriorated the activity of the catalyst but, at the same time, improved its stability. Also, Cao et al. [46] showed that adding Fe to the copper-based catalysts effectively improves their stability in the SRM process.

Because the copper active phase crystallites of all of the catalysts were much better exposed after reduction at 400 °C than at 260 °C, the initial methanol conversation should be higher under SRM conditions for the catalysts which were pre-activated at 260 °C. Indeed, this pattern is observed, but only for the Cu/CeO_2_ catalyst. In the case of the Cu-Fe/CeO_2_ catalysts, higher initial methanol conversion was found after their reduction at 400 °C than at 260 °C. This probably means that the significantly better initial activity of the Cu-Fe systems in the SRM process occurs when the iron is in the Fe_2_O_3_ rather than the Fe^0^ form (Figure 3).

**Figure 7:**
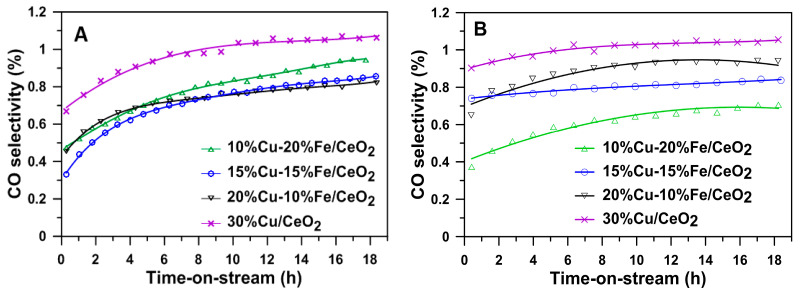

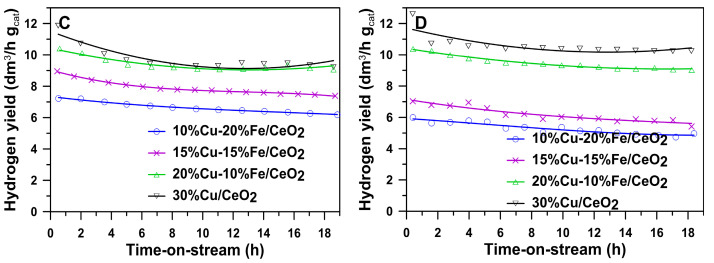
CO selectivity and hydrogen yield on the Cu/CeO_2_ and Cu-Fe/CeO_2_ catalysts reduced at (**A**,**C**) 260 °C and (**B**,**D**) 400 °C in the SRM at 260 °C.

The increase in the iron and decrease in copper content in the active phase of the studied catalysts was also associated with a decrease in the selectivity of these systems to carbon monoxide (Figure 7A,B). This may indicate that iron activates the WGS (3) reaction. Conversely, an increase in iron and a decrease in copper content decreases the most desirable reaction product, hydrogen (Figure 7C,D).

### 2.7. TEM Studies of Cu-Fe Catalysts after Reaction

**Figure 8:**
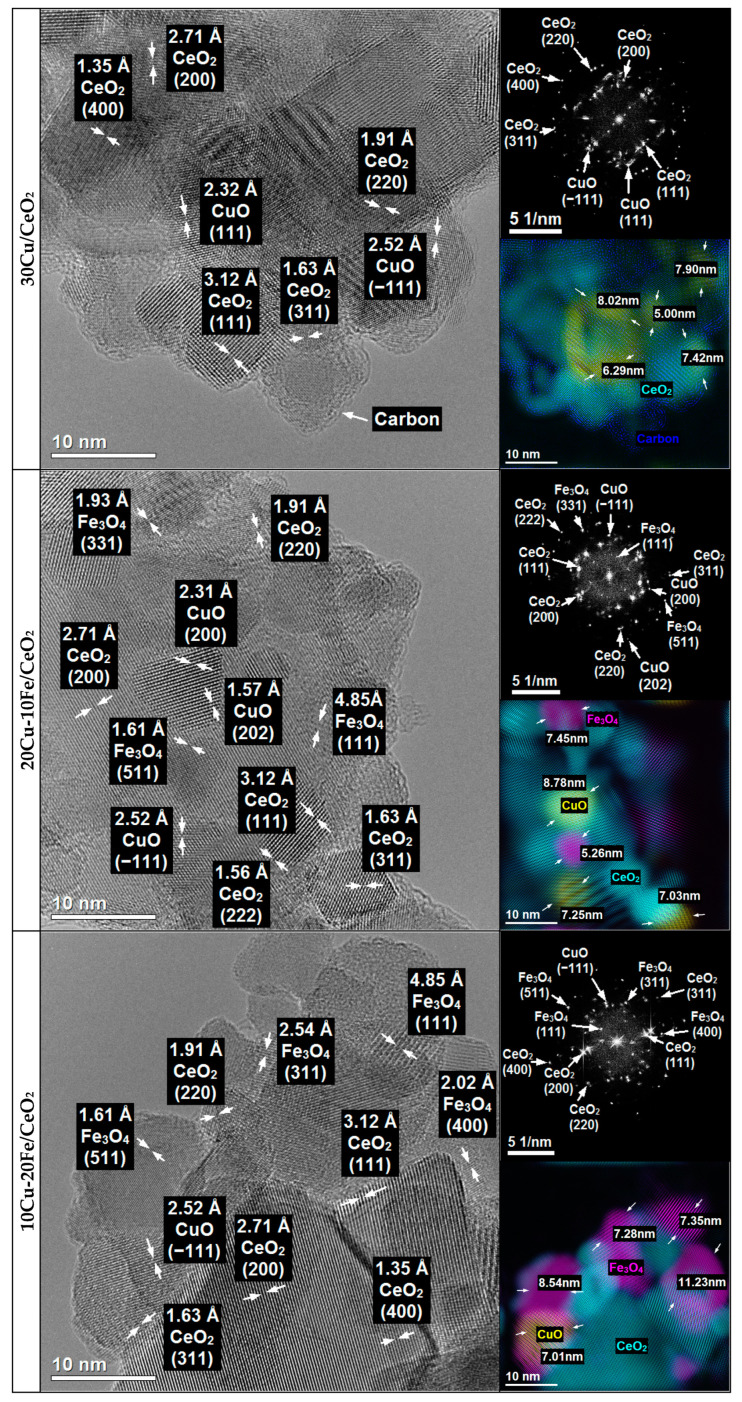
HRTEM images and FFT with phase identification of 30Cu/CeO_2_, 20Cu-10Fe/CeO_2_ and 10Cu-20Fe/CeO_2_ catalysts reduced at 260 °C and after reaction at 260 °C.

**Figure 9:**
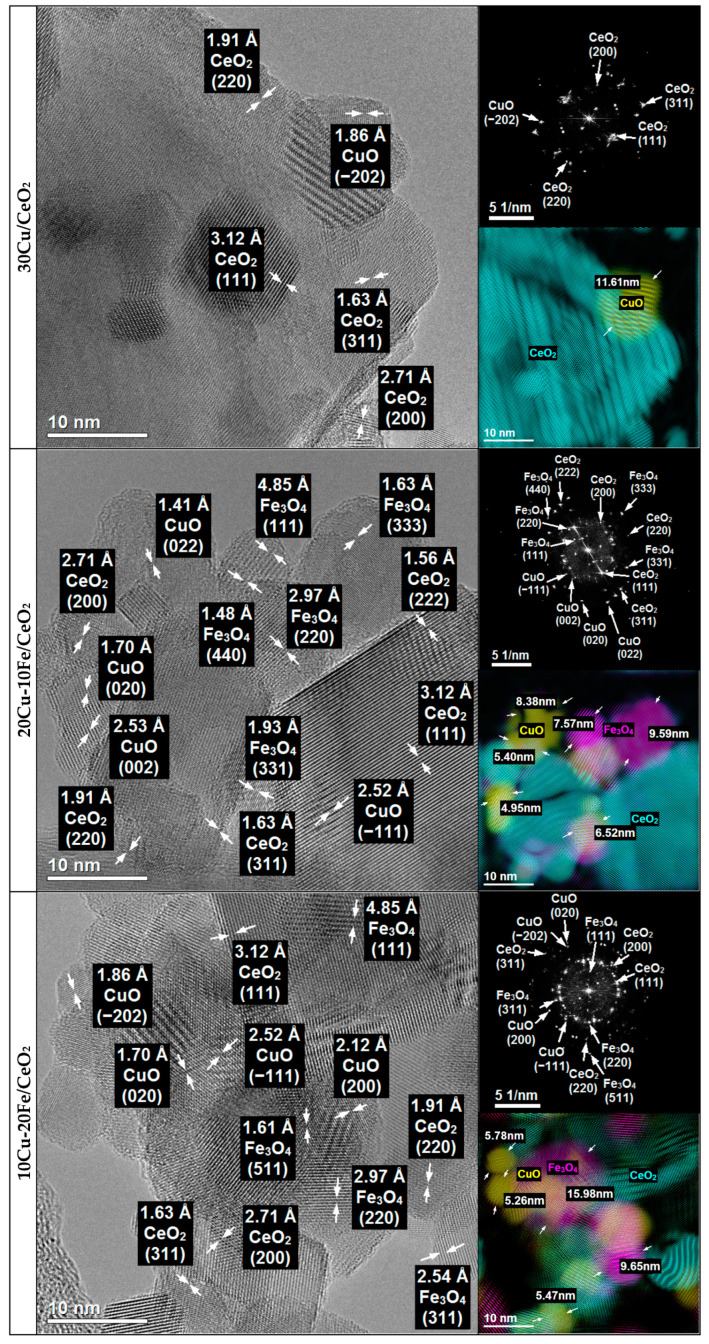
HRTEM images and FFT with phase identification of 30Cu/CeO_2_,20Cu-10Fe/CeO_2_ and 10Cu-20Fe/CeO_2_ catalysts reduced at 400 °C and after reaction at 260 °C.

The TEM and STEM-EDS studies of the 30Cu/CeO_2_ catalyst after 18 h of the steam reforming of methanol reactions are shown in Figure 8, Figure 9, Figure S5 and Figure S6. Depending on the reduction temperature, differences in the location of Cu crystallites on the support surface, the size of Cu crystallites and the amount of carbon deposit on the surface of the catalyst are observed.

In the case of all of the catalysts, the CeO_2_ phase was identified based on the interplanar distances of 3.12 Å, 2.71 Å, 1.91 Å, 1.63 Å, 1.56 Å and 1.35 Å, which corresponded to the lattice planes (111), (200), (220), (311), (222) and (400), respectively (Figure 8 and Figure 9).

The nature of the copper species after the SRM reaction did not depend on the samples’ pre-activation temperature or qualitative and quantitative composition. The copper active phase was identified as CuO based on the interplanar distances of 2.53 Å, 2.52 Å, 2.32 Å, 2.31 Å, and 1.86 Å, 1.70 Å, 1.57 Å and 1.41 Å, which corresponded to the lattice planes (002), (−111), (111) and (200), and (−202), (020), (202) and (022), respectively (Figure 8 and Figure 9). Moreover, in the case of both Cu-Fe/CeO_2_ samples, the Fe_3_O_4_ phase in the catalyst was most often identified based on the interplanar distances of 4.85 Å, 2.97 Å, 2.54 Å, 2.02 Å, 1.93 Å, 1.61 Å and 1.48 Å, which corresponded to the lattice planes (111), (220), (311), (400), (331), (511) and (440), respectively (Figure 8 and Figure 9). Given that copper is predominantly metallic after reduction at 260 and 400 °C, iron is present as Fe_2_O_3_ and/or CuFe_2_O_4_ spinel (for a reduction temperature of 260 °C) or Fe (for a reduction temperature of 400 °C), depending on the reduction temperature, and the presence of the CuO and Fe_3_O_4_ phases after the SRM reaction suggests that the catalysts’ deactivation could be caused by both the copper phase and the iron phase oxidation under the reaction conditions. However, it cannot be excluded that both phases could be oxidised during the transport of the samples from the reduction reactor to the electron microscope in the air.

The distribution of copper and iron species on the surface of the CeO_2_ support after the SRM is similar for all of the catalysts to that obtained after their pre-activation, respectively, at a temperature of 260 °C (Figure S5) or 400 °C (Figure S6). Considering the possible measurement error during the determination of the crystallite size, the crystallite size of CuO and Fe_3_O_4_ (or CuFe_2_O_4_ spinel) of none of the catalysts tested changed under the SRM reaction conditions, and these sizes are comparable to those obtained for the catalysts after reduction at the corresponding temperature (Table 2). Moreover, similar to the results obtained for the samples after reduction at the corresponding temperature (Figure 4 and Figure 5), after the SRM process, the CuO crystallites of the catalysts, pre-reduced at 260 °C, remained mainly in the bulk of the support (Figure 8). In contrast, the CuO crystallites of the catalysts, pre-reduced at 400 °C, even after 18 h of work under SRM conditions, maintained a much better exposure to the copper lattice planes, remained quite well visible on the surface of the support and remained uncovered by the CeO_2_ support (Figure 9).

Deactivation of the Cu/CeO_2_ and Cu-Fe/CeO_2_ catalysts under SRM conditions may also have resulted from forming a carbon deposit on the surface. The amount of carbon deposition formed depended on the catalysts’ pre-activation temperature in a hydrogen atmosphere. Both TEM studies (Figure 8 and Figure 9) and STEM-EDS chemical analysis (Figures S5 and S6) obtained for all of the catalysts show that a significant amount of carbon deposits were formed on the surface of all of the catalysts after their reduction at 260 °C. Meanwhile, carbon formation was not observed, and the production was negligible after the catalysts’ reduction at 400 °C. The carbon deposit might be mainly formed on the catalysts by the Boudouard reaction according to the following equation: 2CO → C + CO_2_ [40,63].

## 3. Materials and Methods

### 3.1. Catalyst Preparation

The Cu-based catalysts containing 15, 30 and 45 wt.% of Cuand the Cu-Fe/CeO_2_ catalysts containing 10, 15 and 20 wt.% Fe and 20, 15 and 10 wt.% Cu, respectively, were prepared by the impregnation of the CeO_2_ support. Before the impregnation, the CeO_2_ support (Sigma-Aldrich, Darmstadt, Germany) was dried at 110 °C for one hour. For the impregnation of the support, aqueous solutions containing the appropriate amount of Cu(NO_3_)_2_·3H_2_O, Fe(NO_3_)_3_·9H_2_O and citric acid CA (Cu + Fe/CA = 1/1 mol/mol) were used. The catalytic systems were dried at 110 °C for 12 h, and the next step was the calcination obtained at 400 °C for 2 h at a heating rate of 2 °C/min. The fine powder, with particle sizes ranging from 0.15 mm to 0.30 mm, was obtained by crushing the calcined pellet.

### 3.2. Catalyst Characterisation

The catalysts were characterised using various techniques, which are briefly outlined below.

#### 3.2.1. Low-Temperature Adsorption of Nitrogen

The low-temperature adsorption of nitrogen on the catalysts was carried out at the temperature of liquid nitrogen (−196 °C) using automatic equipment for the physical adsorption, the ASAP 2420MP (Micromeritics, Norcross, USA). Before the nitrogen adsorption measurements, the catalyst samples were evaporated at 200 °C. The size of the specific surface area of the catalysts was determined by the Brunauer–Emmett–Teller (BET) method. The Barre–Joyner–Halenda (BJH) method was used to determine the mean pore size and volume based on the data obtained from the nitrogen desorption isotherm.

#### 3.2.2. XRF Measurements

The quantitative composition of the element in the copper and copper–iron catalysts was determined by X-ray fluorescence spectroscopy (EDXRF—Energy-Dispersive X-ray Fluorescent). The research was carried out using a Canberra-Packard 1510 spectrometer equipped with a Si(Li) detector cooled with liquid nitrogen. Deconvolution of the spectral data and calculations of the element content in the catalysts were performed using the AXIL system 100 v3.0 software package.

#### 3.2.3. XRD Measurements

X-ray diffraction (XRD) was used to determine the volume phase composition of the fresh and reduced catalysts. X-ray diffractograms were recorded at temperatures of 20 °C, 260 °C and 400 °C using an Empyrean X-ray diffractometer (PANalytical, Malvern, UK) with a CuKα lamp (λ = 1.54 × 10^−10^ m). Before the XRD measurements of the activated catalysts, the samples were reduced in situ at 260 and 400 °C under a hydrogen flow of 100 mL/min in an XRK 900 reaction chamber (Anton Paar, Graz, Austria ). The XRD patterns were collected in the 2θ range from 20° to 110°.

#### 3.2.4. H_2_-TPR Measurements

The temperature-programmed reduction (H_2_-TPR) of the copper–iron catalysts was performed in an AutoChem II 2920 (Micromeritics, Norcross, GA, USA) apparatus equipped with a quartz tubular flow reactor and a thermal conductivity detector (TCD). An amount of 50 mg of catalyst with a grain size of 0.15–0.3 mm was used for the measurements. Before the H_2_-TPR, the samples were pre-treated in a mixture of 5% O_2_ in He. The gas used for the reduction was a mixture of 5% H_2_ in Ar flowing through the reactor at a rate of 30 cm^3^/min. The heating rate was 10 °C/min.

#### 3.2.5. TEM Measurements

Fresh catalysts, catalysts after reduction at 260 °C and 400 °C and catalysts after reaction were subjected to microscopic examination. The catalyst reduction was carried out in a fixed-bed reactor with a hydrogen flow rate of 100 mL/min. The catalysts were ground in an agate mortar into fine powders. The resulting powder of each catalyst was poured into 99.8% ethanol (POCH, Gliwice, Poland) to form a slurry, which was then subjected to ultrasonic homogenisation for 10 s. The suspensions of the catalyst powders in ethanol (99.8%, POCH, Gliwice, Poland) were applied to 200-mesh nickel grids covered with carbon-stabilised lace formvar (Ted Pella Company, Redding, CA, USA) and left on a filter paper until the ethanol evaporated. The nickel grids with the applied samples were placed in a single-tilt holder and transferred to the electron microscope.

The analysis of catalyst samples was performed using a high-resolution electron microscope, the Titan G2 60–300 kV (FEI company, Eindhoven, the Netherlands ). The main equipment of the microscope included a field emission gun (FEG), a monochromator, a three condenser lens system, an objective lens system, image correction (Cs-corrector), an HAADF detector and an EDS (Energy-Dispersive X-Ray Spectroscopy) spectrometer with a Si(Li) detector. Microscopic measurements of the samples were performed at an accelerating voltage of 300 kV.

The element mapping of the catalysts was conducted in STEM mode, collecting EDS spectra step-by-step from each pixel in the map. Each of the collected elemental maps was presented as a coloured matrix of pixels, where in the intensity corresponded to the amount of the element in the mapping place of the sample.

The phase separations (ceria support, and copper and iron active phases) and the identification of various interplanar distances and lattice planes in the samples were carried out based on the FFT (fast Fourier transform) and HRTEM images using Gatan Digital Micrograph software version 1.90.719.

#### 3.2.6. Catalytic Activity Measurement

The steam reforming of the methanol (SRM) reaction was carried out in the temperature range of 180–420 °C, and isothermally at 260 °C at an atmospheric pressure in a quartz fixed-bed reactor with a continuous flow of the reaction mixture. An amount of 0.1 g of the catalyst was used with a grain size of 0.15–0.30 mm, mixed in a weight ratio of 1/10 with quartz with a grain size of 0.15–0.30 mm. The reaction mixture vapour flow rate was 50 mL/min (the molar ratio of the methanol and water vapours was 2/3 mol/mol). The reaction mixture of the water and methanol was introduced into the micromixer using a high-pressure pump (HPLC). The mixture was then introduced into a microchannel evaporator located inside of the heated zone of the reaction system. Next, the reaction mixture was diluted with nitrogen (the flow rate was 50 mL/min). Reaction products were analysed online using two gas chromatographs. The first of them, the Varian 450-GC, equipped with a packed column (Porapaq Q, 2m × 1/8″) and TCD and FID detectors, was used to analyse the water and methanol. The carrier gas in this system was helium. The second chromatograph, the Varian Micro GC CP-4900, used four independent micromodules (channels). The first channel (MS5A molecular sieves) with a TCD detector was supplied with argon as a carrier gas. This channel was used to determine the concentration of hydrogen. Channels two (molecular sieves MS5A), three(PPQ) and four (Al_2_O_3_) were powered by helium. The second channel was used to determine the concentration of nitrogen, methane and carbon monoxide. The third channel was used to determine the concentration of carbon dioxide. The fourth channel, on the other hand, was used to analyse the concentrations of different hydrocarbons.

The total methanol conversion (X_MeOH_, %) and individual carbon-containing products ($S_{C_{i}}$, %) were calculated from the formulas given below:

$$X_{MeOH}=\frac{C_{MeOH}^{in}-C_{MeOH}^{out}}{C_{MeOH}^{in}}\cdot 100\%$$

where

$C_{MeOH}^{in}$—the molar concentration of methanol in the reaction mixture, mol%;
$C_{MeOH}^{out}$—the molar concentration of methanol in the post-reaction mixture, mol%.

$$S_{C_{i}}=\frac{n_{i}C_{i}^{out}}{C_{CO}^{out}+C_{CO_{2}}^{out}+C_{CH_{4}}^{out}}\cdot 100\%$$

where

$C_{i}^{out}$—the molar concentration of carbon-containing products in the post-reaction mixture, mol%;
$n_{i}$—the number of carbon atoms in the molecules of the post-reaction carbon products.

The selectivity of the hydrogen formation ($S_{H_{2}}$, %) and hydrogen yield (Y, dm^3^/h∙g_cat_) were determined from the following equations:

$$S_{H_{2}}=\frac{C_{H_{2}}^{out}}{3\cdot (C_{CO}^{out}+C_{CO_{2}}^{out}+C_{CH_{4}}^{out})}\cdot 100\%$$

where

$C_{H_{2}}^{out}$—the molar concentration of the hydrogen in the post-reaction mixture, mol%.

$$Y=\frac{3V_{m}G_{\mathrm{Me}}X_{\mathrm{MeOH}}S_{H_{2}}}{10^{4}m_{c}}$$

where

*G*_Me_—the initialmolar flow rateof methanol (mol/h);
*V*_m_—the molar volume (dm^3^/mol); *m*_c_—the sample weight [64].

## 4. Conclusions

The results presented in this paper show how the composition, reduction temperature, morphology, structure and oxidation state of the copper–iron active phase affected the activity, selectivity and stability of the Cu-Fe/CeO_2_ systems in the steam reforming of methanol. The presence of iron in the Cu/CeO_2_ catalyst improved the stability of this system and, at the same time, reduced the production of undesirable CO in the SRM. On the other hand, the amount of the copper active phase influenced the activity of the catalysts, and 30 wt.% was optimal for maintaining the high methanol conversion. Therefore, alcohol conversion decreased with the decrease in the amount of copper.

An increase in the reduction temperature from 260 °C to 400 °C resulted in an improvement in the methanol conversion over the 30Cu/CeO_2_ system and a worsening in the methanol conversion over the Cu-Fe/CeO_2_ systems. In the case of the Cu/CeO_2_ catalyst, the better exposure of the copper active phase crystallites after reduction at 400 °C than at 260 °C is probably responsible for the much higher initial activity of this catalyst after its pre-activation at 400 °C than at 260 °C. However, in the case of the Cu-Fe/CeO_2_ catalyst, the oxidation form of iron seems to have a higher influence on the initial methanol conversion. Therefore, significantly better activity of the Cu-Fe/CeO_2_ systems in the SRM process occurred when the iron was in the Fe_2_O_3_ form after reduction at 260 °C than in the Fe^0^ form after reduction at 400 °C.

The higher 30Cu/CeO_2_ catalyst reduction temperature (400 °C) also increased the size of the copper crystallite. It provided better exposure of the lattice planes of the copper active phase crystallites to the reaction reactants. This, in turn, significantly impacted the improvement of the activity of this system in the SRM. In the case of the bimetallic Cu-Fe/CeO_2_ catalysts, the higher reduction temperature did not cause the sintering of the crystallites of the copper active phase. The reason for this was the presence of iron in these systems, which prevented the movement of the copper crystallites on the CeO_2_ support surface due to the immiscible interaction between Cu and Fe, where the iron compounds can suppress the sintering of the copper particles.

The analysis of all of the studied catalysts after the SRM process showed that the deactivation of the studied catalysts under SRM conditions could result from the oxidation of both the copper phase and the iron phase and/or from the formation of the carbon deposits on their surface due to the Boudouard reaction. The oxidation of the copper and iron phases during the SRM process was observed regardless of the pre-activation temperature. However, significant carbon deposits were found for only the catalysts pre-reduced at 260 °C. After a reduction at 400 °C, the amount of carbon deposit was relatively negligible.

## Figures and Tables

**Table 1:** Active metal content, specific surface area and pore size in the Cu-Fe/CeO_2_ catalysts determined based on the XRF analysis ^1^ and the low-temperature nitrogen adsorption ^2^.

Catalyst	Metal Content (wt.%) ^1^		Surface Area [m^2^/g] ^2^	Pore Size [nm] ^2^
	Cu	Fe		
CeO_2_	-	-	63.9	15.4
30Cu/CeO_2_	31.0	-	44.3	14.0
20Cu-10Fe/CeO_2_	20.6	8.9	53.4	11.8
15Cu-15Fe/CeO_2_	16.5	14.3	51.5	13.1
10Cu-20Fe/CeO_2_	11.6	21.1	65.1	10.9

**Table 2:** Crystallite sizes of the copper and iron phases in the fresh Cu-Fe/CeO_2_ catalysts after reduction and after reaction in the steam reforming of methanol.

Form	Phase	Catalyst		
		30Cu/CeO_2_	20Cu-10Fe/CeO_2_	10Cu-20Fe/CeO_2_
		Crystallite Size [nm]		
Fresh	CuO	6.16–9.73	5.52–8.31	7.86
	Fe_2_O_3_	-	6.85	8.52
	Fe_3_O_4_/CuFe_2_O_4_	-	6.57	8.15
Reduced at 260 °C	Cu_2_O	5.82–6.85	3.41–4.42	6.45
	CuO	-	4.22–6.95	4.07–4.67
	Fe_3_O_4_/CuFe_2_O_4_	-	5.75–6.56	6.26–14.12
Reduced at 400 °C	Cu_2_O	13.86–14.94	4.13	7.14
	CuO	-	5.08–6.64	4.95–5.83
	Fe_3_O_4_/CuFe_2_O_4_	-	4.18–7.81	8.01–15.09
After reaction reduced at 260 °C	CuO	5.00–8.02	7.03–8.78	7.01
	Fe_3_O_4_/CuFe_2_O_4_	-	5.26–7.45	7.28–11.23
After reaction reduced at 400 °C	CuO	11.61	4.95–8.38	5.26–5.78
	Fe_3_O_4_/CuFe_2_O_4_	-	7.57–9.59	9.65–15.98

## Data Availability

Data are contained within the article and Supplementary Materials.

## Supplementary Materials

The following supporting information can be downloaded at: https://www.mdpi.com/article/10.3390/molecules29163963/s1, Figure S1: HRTEM images and FFT with phase identification of fresh 30Cu/CeO_2_, 20Cu-10Fe/CeO_2_ and 10Cu-20Fe/CeO_2_ catalysts; Figure S2: STEM-EDS analysis of fresh Cu-Fe/CeO_2_ catalysts; Figure S3: STEM-EDS analysis of Cu-Fe/CeO_2_ catalyst reduced at 260 °C; Figure S4: STEM-EDS analysis of Cu-Fe/CeO_2_ catalyst reduced at 400 °C; Figure S5: STEM-EDS analysis of Cu-Fe/CeO_2_ catalyst reduced at 260 °C and after reaction at 260 °C; Figure S6: STEM-EDS analysis of Cu-Fe/CeO_2_ catalyst reduced at 400 °C and after reaction at 260 °C.
