# Supplementary material for: Hydrogen Production from Methanol Steam Reforming over Fe-Modified Cu/CeO2 Catalysts
Source: Molecules. 2024 Aug 22;29(16):3963. doi: 10.3390/molecules29163963 (PMC11357062; doi:10.3390/molecules29163963)
Supplement: Supplementary file 1 [file molecules-29-03963-s001.zip › molecules-3105519-supplementary.pdf]

Hydrogen production from methanol steam reforming over Fe-modified Cu/CeO_2 catalysts

Authors (name and surname): Grzegorz Słowik, Marek Rotko, Janusz Ryczkowski, Magdalena Greluk

Affiliation: Department of Chemical Technology, Institute of Chemical Sciences, Faculty of Chemistry, Maria Curie-Sklodowska University in Lublin, Poland

Corresponding author: Grzegorz Słowik, Department of Chemical Technology, Institute of Chemical Sciences, Faculty of Chemistry, Maria Curie-Sklodowska University in Lublin, 3 Maria Curie-Skłodowska Square, 20-031 Lublin, Poland,

E-mail: grzegorz.slowik@mail.umcs.pl

3.3.2.1 TEM studies of fresh Cu-Fe catalysts

Figure S1 shows the phase analysis in the fresh Cu-Fe/CeO_2 catalysts using a transmission electron microscope. A significant part of the catalyst's composition is the CeO_2 support, characterised by crystallites of various shapes and sizes. The remaining part of the catalysts consisted of fine, circular, relatively evenly dispersed crystallites of the copper–iron active phase. The phase identification based on the HRTEM and FFT images allowed for the separation of individual phases in the studied fragments of the catalysts. In this way, in all catalysts, the phase originating from the CeO_2 support was identified mainly based on the interplanar distances 3.12 Å, 2.71 Å, 1.91 Å, 1.63 Å, 1.56 Å, assigned, respectively, to the following lattice planes: (111), (200), (220), (311) and (222). These parameters correspond to the cubic crystallographic system. The analysis of the active phase in the 30Cu/CeO_2 catalysts showed the presence of copper in the copper oxide form (CuO), identified based on interplanar distances of 2.52 Å and 2.32 Å, which corresponds to the lattice plane (−111), (111), respectively, and monoclinic crystallographic system. The crystallite size of CuO in the catalyst has been estimated at 6.16 – 9.73 nm (Table 2).

In the case of the 20Cu-10Fe/CeO_2 catalyst with the copper–iron active phase, apart from copper in the CuO form, the presence of an active phase in the $CuFe_2O_4$ spinel form with a cubic and tetragonal crystallographic system and iron as a separate phase in the Fe_2O_3 form with the rhombohedral crystallographic system was also noted. CuO was identified based on the interplanar distances of 2.53 Å, 2.32 Å, and 2.31 Å, assigned to the lattice plane (002), (111), and (200), respectively. The $CuFe_2O_4$ spinel in the studied fragment of the catalyst was identified based on interplanar distances of 2.58 Å, 2.54 Å, and 1.92 Å, corresponding to the lattice plane (103), (311), and (331), respectively. Nevertheless, the positions of some peaks and interplanar distances for Fe_3O_4 and $CuFe_2O_4$ are similar, which causes uncertainty in the unambiguous determination of these phases. The Fe_2O_3 contribution is minor due to the small amount of iron in the catalyst, but it was identified by the interplanar distance of 2.69 Å, which corresponds to the lattice plane (104). Phase identification performed for Cu-Fe/CeO_2 catalysts based on the electron microscopy analysis corresponds quite well with the results obtained from XRD and XPS. The crystallite size of CuO in the catalyst was in the range of 5.52–8.31 nm. It was smaller than in the case of the crystallite size determined for the 30Cu/CeO_2 catalyst, which may result from a lower copper content in this system or

an increase in copper dispersion due to the addition of iron to the catalyst. In turn, the sizes of the Fe_2O_3 and $CuFe_2O_4$ spinel crystallites were about 6.85 nm and 6.57 nm, respectively.

The phase composition in the fresh 10Cu-20Fe/CeO_2 catalyst based on HRTEM and FFT imaging was similar to that for the fresh 20Cu-10Fe/CeO_2 catalyst. This system's copper and iron species were identified as CuO, Fe_2O_3, and $CuFe_2O_4$. The CuO phase in the catalyst was determined based on an interplanar distance of 2.52 Å, which corresponded to the (111) lattice plane. The crystallites of this phase reached a size of 7.86 nm and were smaller than in the case of other systems. The Fe_2O_3 phase in the catalyst was identified based on the interplanar distances of 1.84 Å and 1.49 Å, which were assigned to the (024) and (214) lattice planes, respectively. The crystallites of this phase had a size of 8.52 nm. In turn, the $CuFe_2O_4$ spinel (or Fe_3O_4) in the studied system was identified based on interplanar distances of 2.58 Å, 2.54 Å, and 1.59 Å, which corresponded to the (103), (311), and (321) lattice planes, respectively. The crystallites of this phase had a size of 8.15 nm.

Figure S2 shows the STEM image and element distribution on the EDS maps from the selected area of the studied catalysts. The STEM image of the catalysts shows crystallites with various shapes and sizes originating from the ceria support and the copper–iron active phase. The EDS maps show the dispersion of the constituent elements and their position relative to each other in the catalysts. The Cu+Ce map of the 30Cu/CeO_2 catalyst shows that cerium oxide crystallites are primarily copper-covered. The copper clusters in the catalyst are pretty large, sometimes comparable to the crystallites of the CeO_2 support, which results from the formation of copper agglomerates. The STEM-EDS analysis of the fresh bimetallic Cu-Fe/CeO_2 catalysts shows that a significant part of the STEM image are crystallites of the CeO_2 support on which iron and copper crystallites are quite well dispersed (Cu+Fe+Ce map). The copper crystallites in the Cu-Fe/CeO_2 catalysts are smaller than those in the 30Cu/CeO_2 sample, and both copper and iron occur close to each other or even in similar places. In the case of the 20Cu-10Fe/CeO_2 sample, it can also be observed that copper exists on the support separately due to its dominant amount in the catalyst. Meanwhile, in the case of the 10Cu-20Fe/CeO_2 sample, due to the dominant contribution of iron, it exists on the support separately in the form of significant clusters. Furthermore, the presented STEM-EDS studies indicate that with the increase in the iron content and the decrease in the copper content in the active phase of the catalysts, an increase in the dispersion of copper crystallites in the catalysts was observed. This may be due to the decreasing copper content in the catalysts and, consequently, to a decrease in the crystallite size of the copper active phase.

In all studied catalysts, the Cu+Ce and Cu+Fe+Ce EDS maps also show that Cu and Fe crystallites in the catalysts are not very clearly visible, exposed on the surface, but are often covered to some extent by ceria support crystallites.

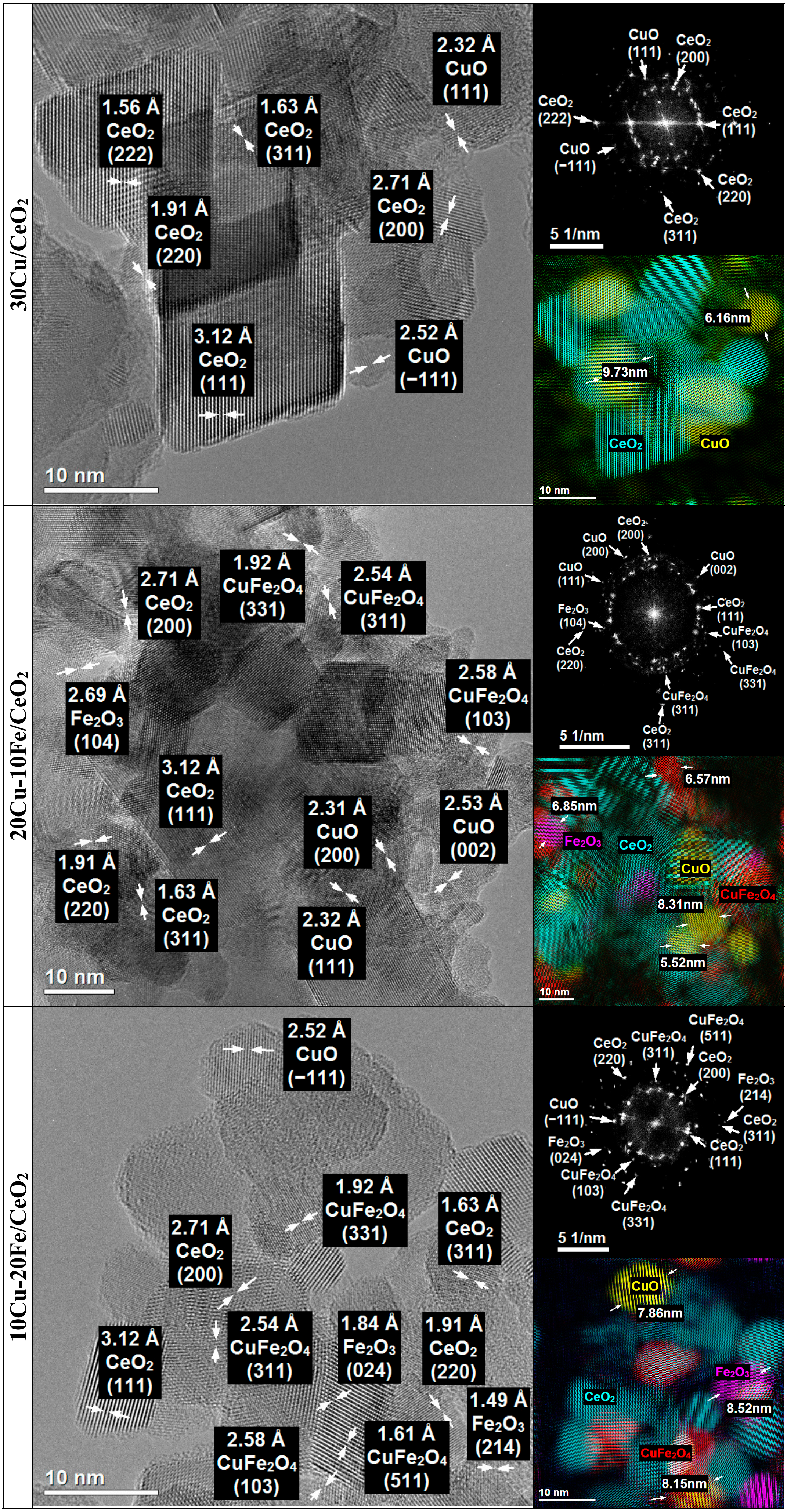

Figure S1. HRTEM images and FFT with phase identification of fresh 30Cu/CeO_2, 20Cu-10Fe/CeO_2, and 10Cu-20Fe/CeO_2 catalysts.

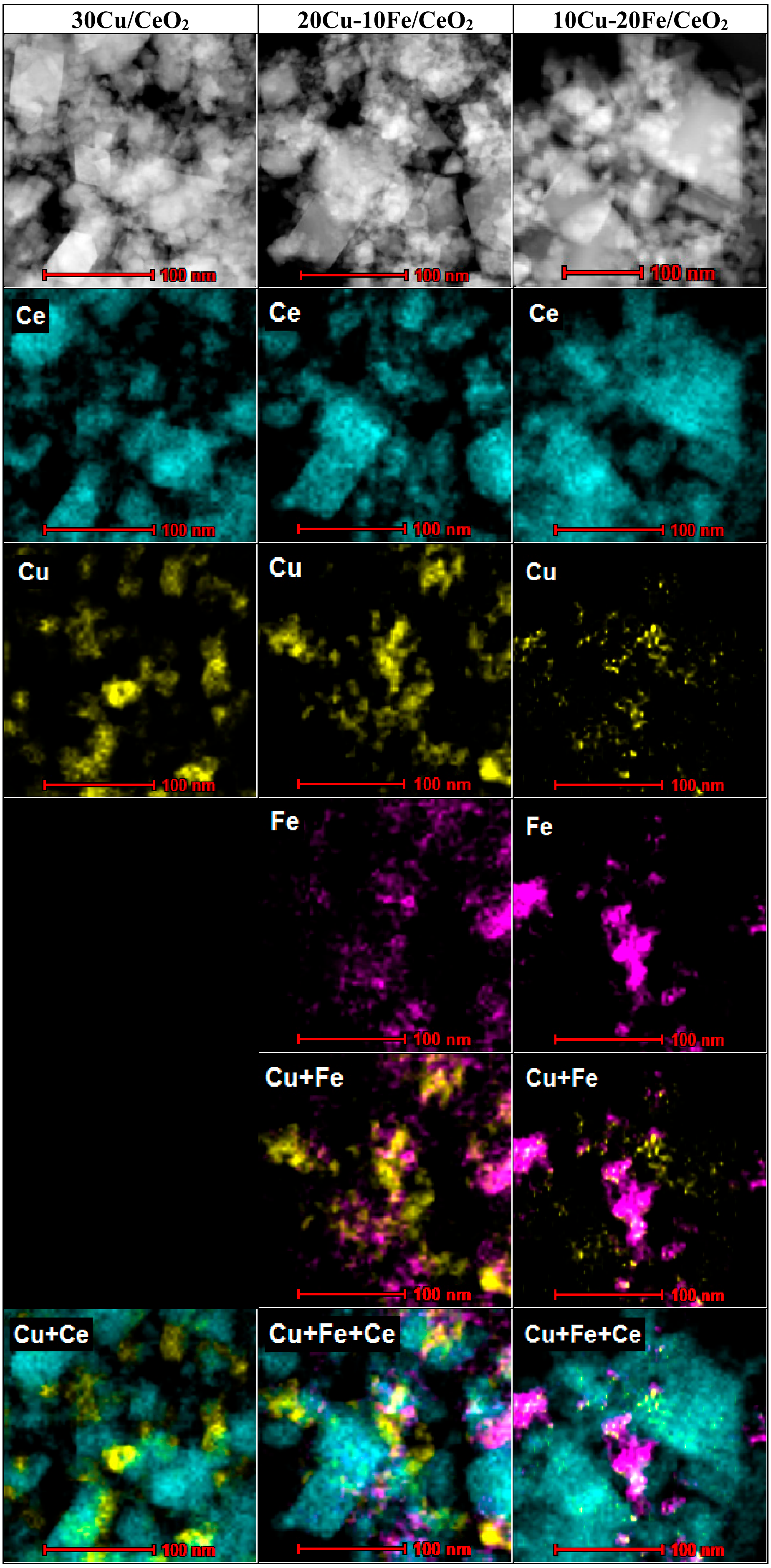

Figure S2. STEM-EDS analysis of fresh Cu-Fe/CeO_2 catalysts.

3.3.2.2 TEM studies of reduced Cu-Fe catalysts

Figure S3 and Figure S4 show STEM-EDS analysis of copper and copper–iron catalysts reduced at 260 °C and 400 °C.

The copper active phase in both monometallic and bimetallic samples after their reduction at 260 ºC and 400 ºC was in the form of Cu_2O, which was identified based on the interplanar distances of 2.98 Å, 2.46 Å, 2.12 Å, and 1.74 Å, corresponding to the lattice planes (110), (111), (200), and (211), respectively. Additionally, the CuO phase in the case of the Cu-Fe catalysts was identified generally based on the interplanar distances of 2.75 Å, 2.52 Å, 1.86 Å, and 1.72 Å, which corresponded to the lattice planes (110), (−111), (−202), and (020), respectively. Moreover, the presence of both copper forms (Cu_2O and CuO) in the Cu-Fe catalysts may result from the different sizes of copper crystallites and the various interactions with the support, which can influence the degree of copper oxidation after the catalysts' contact with atmospheric oxygen. The phase identification for the bimetallic Cu-Fe samples reduced at 260 °C or 400 °C also revealed the nature of the iron species. The Fe3O4 (or CuFe2O4 spinel) phase in the catalysts was identified based on the interplanar distances of 4.85 Å, 2.97 Å, 2.54 Å, 2.09, 1.61 Å, and 1.48 Å, which corresponded to the lattice planes (111), (220), (311), (400), (511), and (440), respectively. Because the STEM-EDS analysis for the 20Cu-10Fe/CeO_2 and 10Cu-20Fe/CeO_2 catalysts after reduction at 260 °C or 400 °C (Figures S3 and S4) shows that in some cases both copper and iron are located in similar places, it may suggest the formation of a $CuFe_2O_4$ spinel after catalyst samples come into contact with atmospheric oxygen during transport and their preparation for TEM studies. Nevertheless, a significant part of both metals of the active phase are present separately on the support, which also results from phase identification.

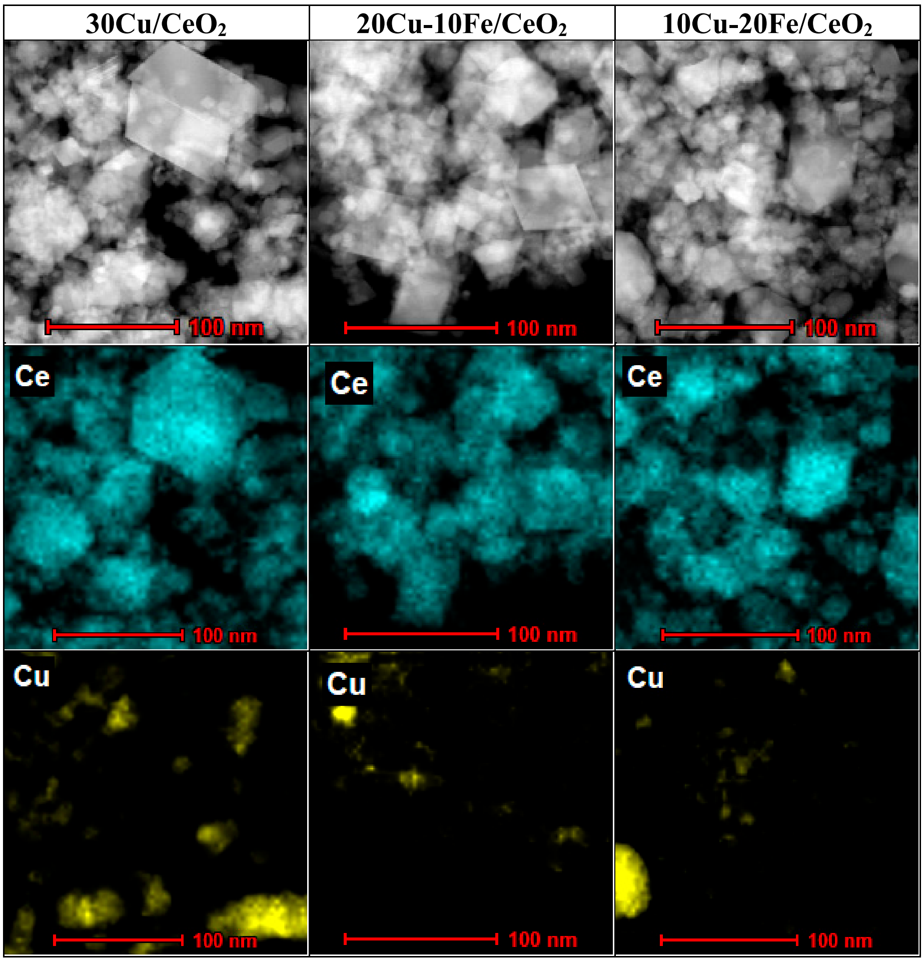

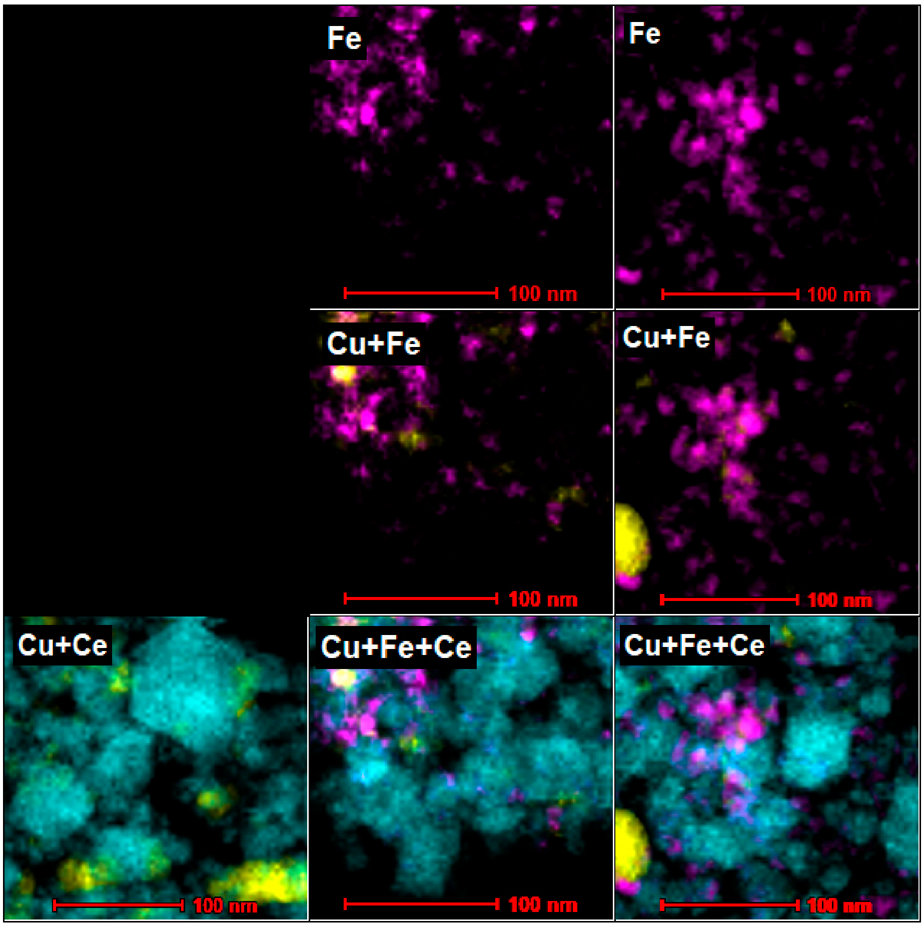

Figure S3. STEM-EDS analysis of Cu-Fe/CeO_2 catalyst reduced at 260 °C.

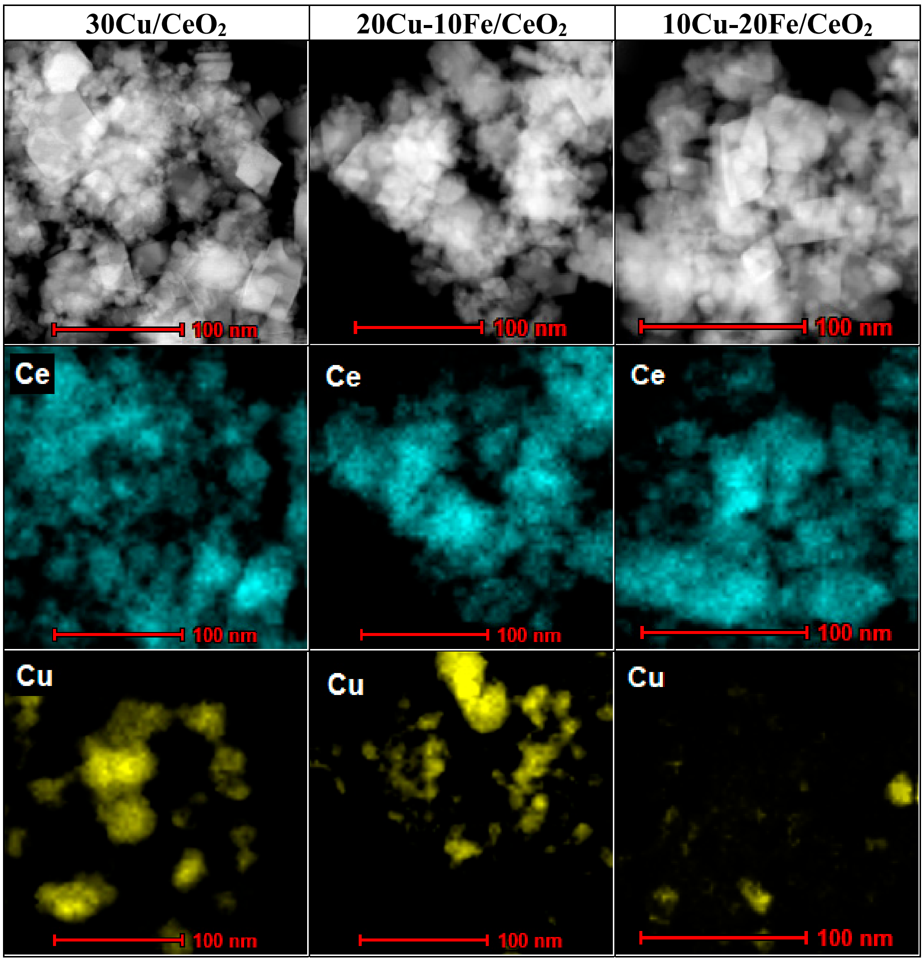

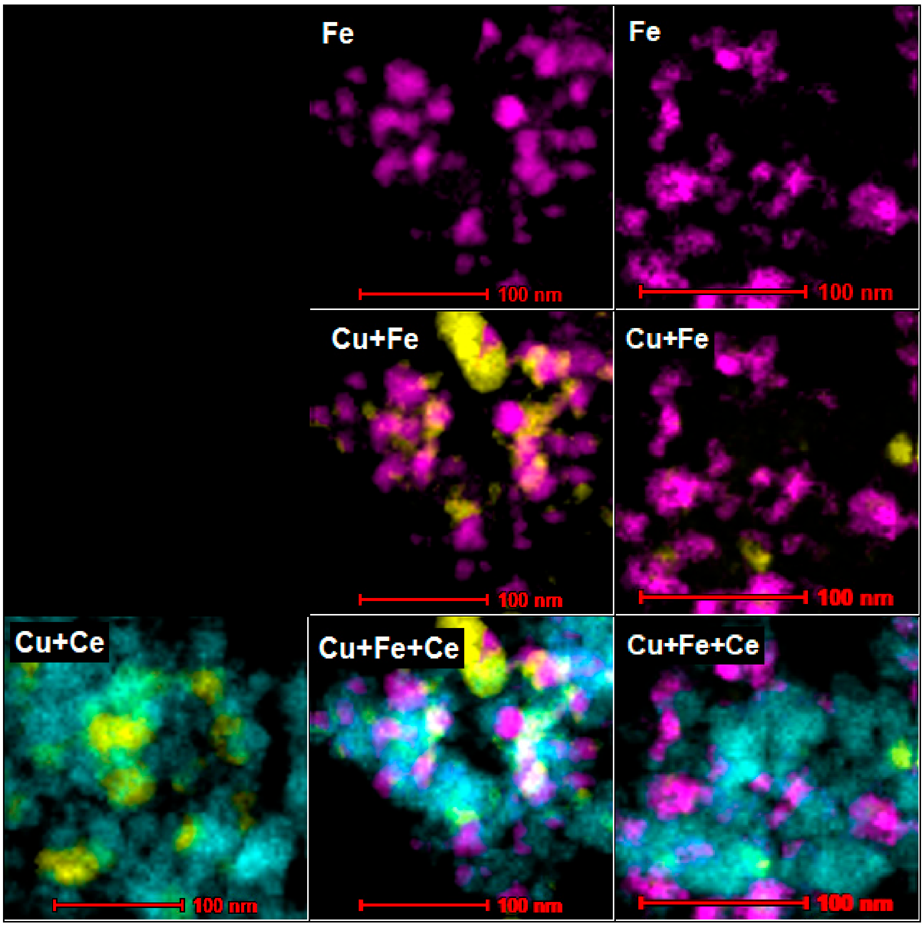

Figure S4. STEM-EDS analysis of Cu-Fe/CeO_2 catalyst reduced at 400 °C.

Figures S3 and S4 show the STEM-EDS analysis of copper and copper–iron catalysts reduced at 260 °C or 400 °C and after 18 h of work at 260 °C in the SRM process.

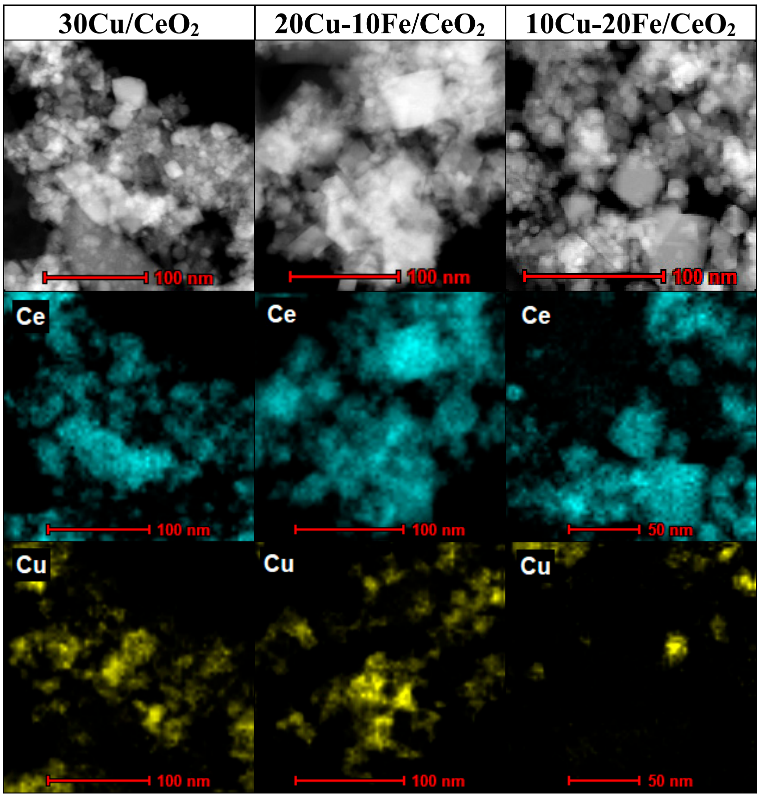

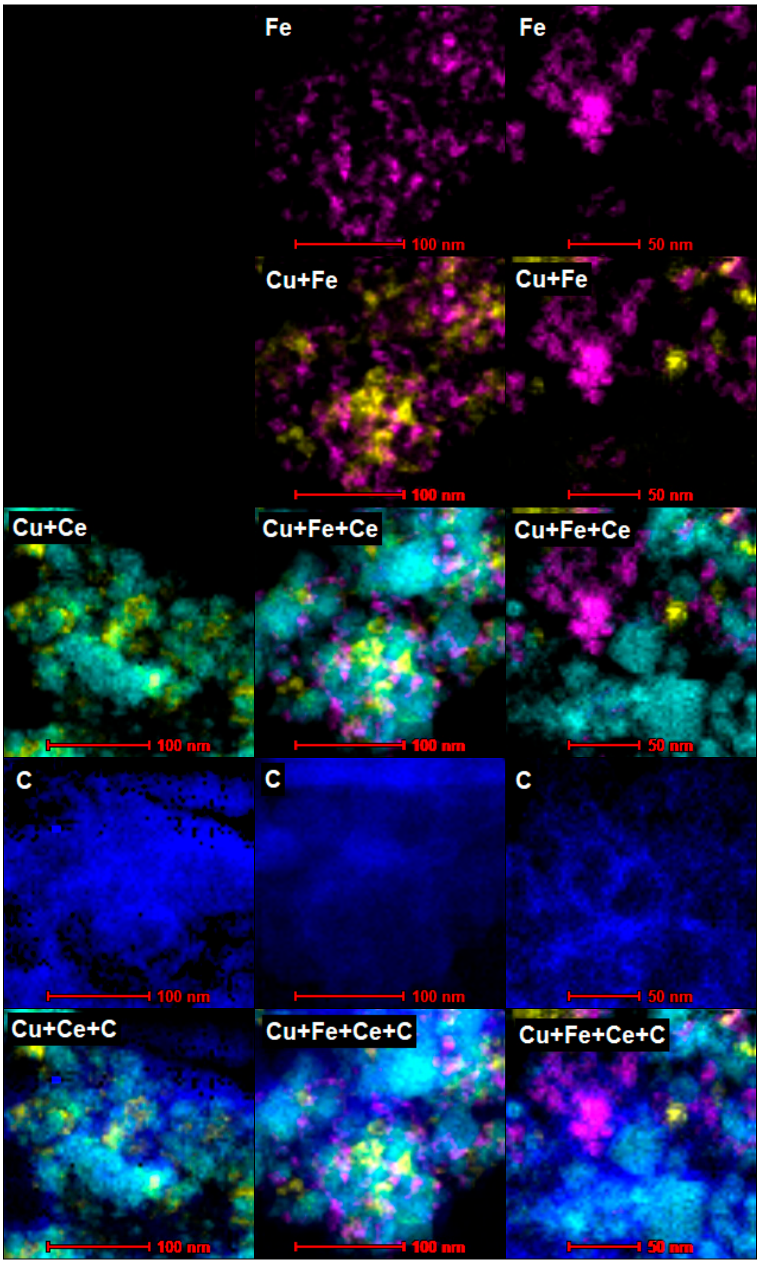

Figure S5. STEM-EDS analysis of Cu-Fe/CeO_2 catalyst reduced at 260 °C and after reaction at 260 °C.

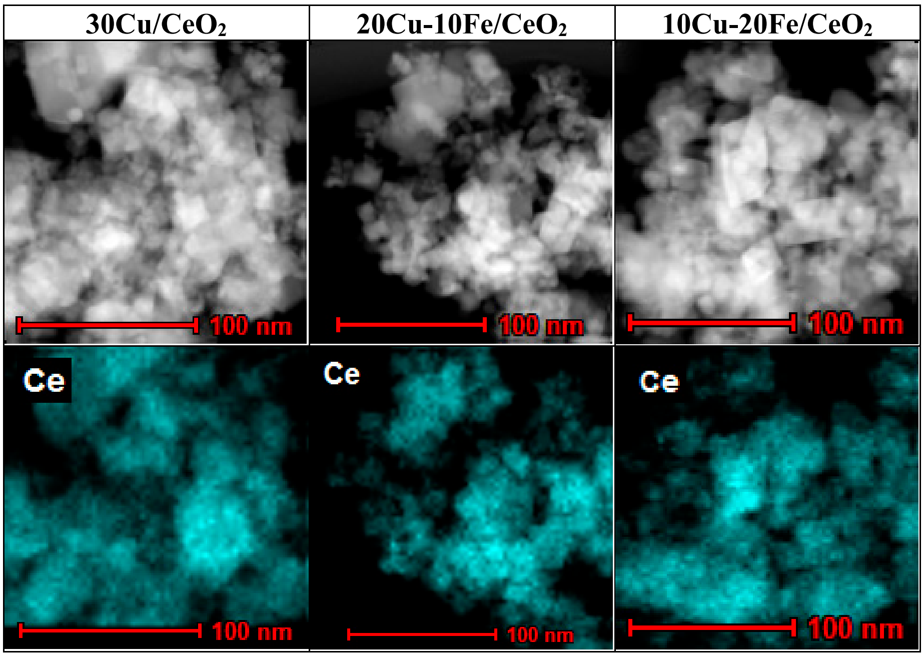

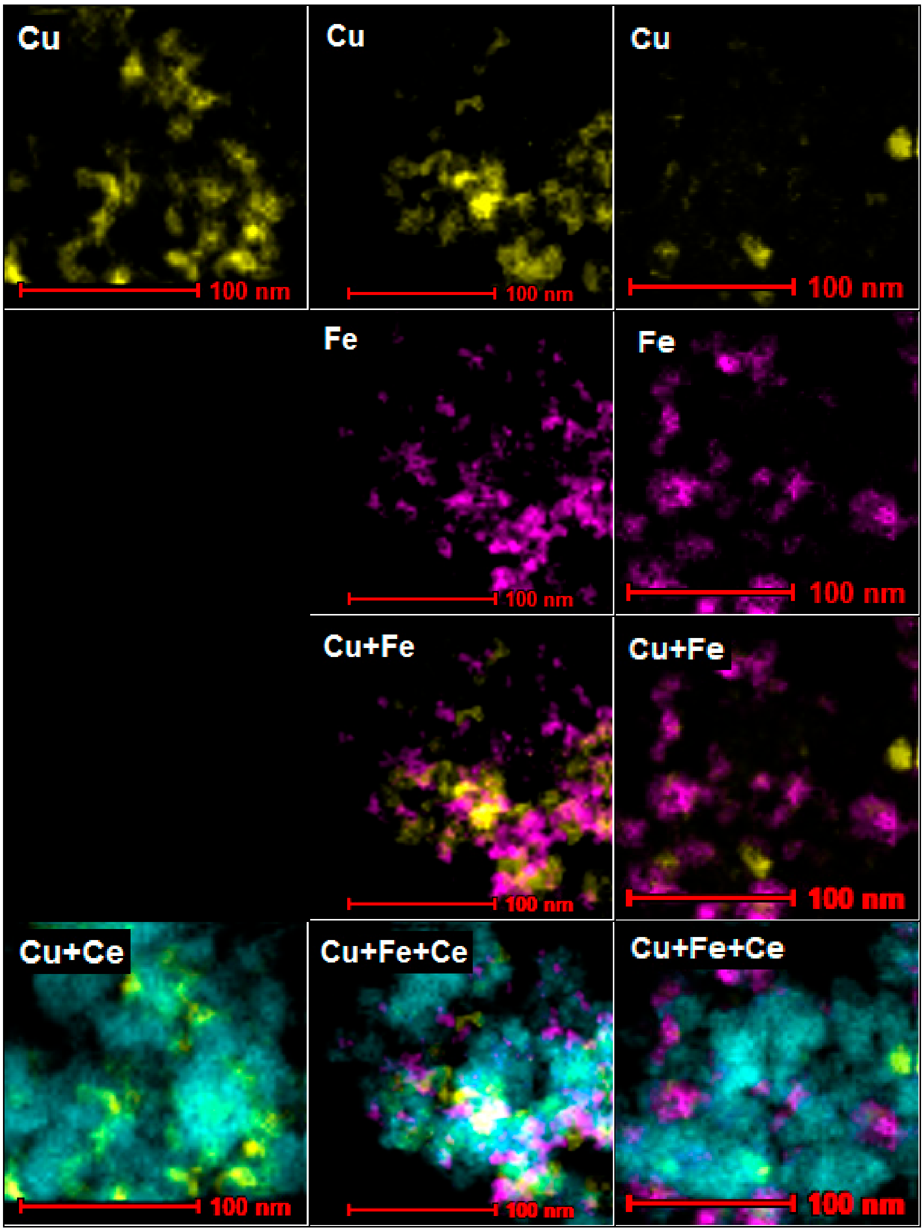

Figure S6. STEM-EDS analysis of Cu-Fe/CeO_2 catalyst reduced at 400 °C and after reaction at 260 °C.
